# PM2.5-Related Burden of Tracheal, Bronchial, and Lung Cancer in China, the United States, Japan, and South Korea (1990–2021)

**DOI:** 10.3389/ijph.2025.1608392

**Published:** 2025-09-09

**Authors:** Ruirui Zhang, Yang Zheng, Zheng Wang

**Affiliations:** ^1^ Department of Staff Healthcare, West China Hospital of Sichuan University, Chengdu, Sichuan, China; ^2^ Department of Nephrology, Nanchong Hospital of Traditional Chinese Medicine, Nanchong, Sichuan, China; ^3^ Office of the Party Committee of West China Clinical Medical College, Sichuan University, Chengdu, Sichuan, China

**Keywords:** PM2.5, tracheal bronchial and lung cancer, disease burden, GBD 2021, household air pollution

## Abstract

**Objectives:**

To quantify the tracheal, bronchial, and lung cancer burden attributable to particulate matter pollution in selected countries, and thereby provide evidence for context - specific public health interventions.

**Methods:**

Based on Global Burden of Disease (GBD) 2021 data,we appliedJoinPoint to analyze the temporal trends from 1990–2021 in the burden of PM2.5 - attributable tracheal, bronchial, and lung (TBL) cancer in China, the US, Japan, and South Korea. And we explored the age, period, cohort effects and predicted future trends using the age-period-cohort mode and Bayesian analysis.

**Results:**

Globally, the age-standardized mortality rate (ASMR) for TBL cancers due to PM2.5 exposure showed a declining trend with an average annual percentage change (AAPC) of-1.2811 from1990 to 2021, projections suggest continued decreases in ASMR in selected countries over the next 29 years, but a global increase is expected.

**Conclusion:**

Studies have demonstrated a global decline in the mortality burden attributed to bronchogenic carcinoma linked to PM2.5. Nevertheless, future projections indicate that the global burden of air pollution-related TBL cancer will rise, effective public health strategies are urgent to develop.

## Introduction

The rapid expansion of the global economy and the acceleration of industrialization have significantly exacerbated air pollution, driven by fossil fuel combustion across transportation, industrial activities, agricultural production, and construction. This widespread pollution now affects almost all of the global population (99%), presenting a substantial threat to public health [1]. Air pollution is responsible for an estimated 7 million premature deaths annually and contributes to over 3% of the global disability-adjusted life years (DALYs) lost. Among the various pollutants, particulate matter (PM) is particularly detrimental due to its ability to induce systemic inflammation and trigger epigenetic alterations, thereby increasing the risk of a range of diseases [2, 3]. Fine particulate matter (PM2.5) is of particular concern, as it is a major contributor to adverse health effects. Sources of PM2.5 include both ambient particulate matter pollution (APM) from industrial emissions, vehicle exhaust, and construction activities, as well as household air pollution (HAP) arising from the incomplete combustion of solid fuels [4]. Numerous studies have established a strong association between PM2.5 exposure and the development and progression of TBL cancers. This link underscores the urgent need to understand the disease burden posed by PM2.5 [5].

China, the United States, Japan, and South Korea—four major global economies with differing levels of industrialization, urbanization, and environmental policies—provide valuable comparative contexts for studying this issue [6, 7]. Rapid economic development in these countries has resulted in significant disease burdens attributable to PM2.5 exposure [8]. Among these, the burden of TBL cancers represents a substantial proportion, imposing severe health and economic challenges [9, 10]. A comprehensive evaluation of the burden of these cancers due to ambient particulate matter pollution in these countries is essential. Such an analysis provides vital insights for formulating effective public health strategies and environmental policies tailored to the specific contexts of each country. Additionally, it enables a comparative analysis of healthcare systems and environmental policies, shedding light on how different socioeconomic and policy environments influence health outcomes.

This study aims to update the assessment of the burden of TBL cancers attributable to PM2.5 exposure from 1990 to 2021. By utilizing data from reliable sources and employing advanced statistical methods, this research seeks to identify trends across genders, regions, and socioeconomic groups. The findings will enhance our understanding of the current health landscape and inform the development of targeted prevention and intervention strategies. Ultimately, this work aims to contribute to global efforts to reduce the burden of these cancers and improve public health outcomes.

## Methods

### Data Sources

The Global Burden of Disease (GBD) 2021 study provides comprehensive epidemiological estimates for 371 diseases and injuries across 204 countries and territories, categorized by age and sex, covering the period from 1990 to 2021. Detailed methodologies have been documented, and both fatal and non-fatal estimates are publicly available (https://vizhub.healthdata.org/gbd-results/) [11]. In the GBD 2021 study, data were systematically collected from surveys, censuses, civil registration systems, demographic surveillance, and other health-related sources. The risk of bias for each data source was evaluated and corrected using DisMod-MR 2.1, a Bayesian meta-regression tool. This study assessed mortality and disability-adjusted life year (DALY) burdens associated with TBL cancers attributable to ambient particulate matter pollution (PM2.5), including household air pollution from solid fuels (HAP) and ambient particulate matter pollution (APM), in China, the United States, Japan, and South Korea. Analyses adhered to GBD protocols and the Guidelines for Accurate and Transparent Health Estimates Reporting (GATHER).

### Statistical Analysis

#### Joinpoint Regression

Joinpoint regression analysis was applied to assess trends in mortality and DALY burdens of tracheal, bronchus, and lung cancers attributable to ambient particulate matter pollution between 1990 and 2021. This method, proposed by Kim in 2000, employs piecewise regression to identify statistically significant trends within distinct segments [12]. Regression fitting was conducted on the natural logarithm of mortality and DALY rates for various segments, and the annual percentage change (APC) along with its 95% confidence interval (CI) was calculated for each period. Trends were summarized at the global, regional, and national levels using the average [13] overlapping 95% CIs and a P-value < 0.05, rejecting the null hypothesis of no variation.

### Age-Period-Cohort Model

The Age-period-cohort model was used to evaluate the effects of age, period, and cohort on health outcomes. The age effect reflects variations in risk across age groups, the period effect captures temporal changes influencing all age groups uniformly, and the cohort effect accounts for differences among individuals born in the same time period [13]. The log-linear regression model was formulated as:

$$\log\left(\text{Yi}\right)=\mu +\alpha \times \text{agei}+\beta \times \text{periodi}+\gamma \times \text{cohorti}+\varepsilon$$

Where Yi represents the mortality or DALY rate of TBL cancers attributable to PM2.5 pollution, α, β, and γ are coefficients for age, period, and cohort effects, respectively, μ is the intercept, and ε denotes residual error. The intrinsic estimator (IE) method was used to calculate the net effects for the three dimensions.

### Bayesian Age-Period-Cohort (BAPC) Analysis

A Bayesian age-period-cohort (BAPC) analysis was conducted using the BAPC and INLA packages in R to predict age-standardized rates (ASR) from 2022 to 2050 by sex. The BAPC model employs an integrated nested Laplacian approximation to derive marginal posterior distributions, addressing mixing and convergence issues associated with the Markov Chain Monte Carlo technique traditionally used in Bayesian methods [14].

All statistical analyses and visualizations were performed using R (version 4.2.2). Differences were considered statistically significant at a two-sided P-value < 0.05.

## Results

### TBL CancerS Burden Trends Attributable to Ambient PM Pollution (1990–2021)

**FIGURE 1:**
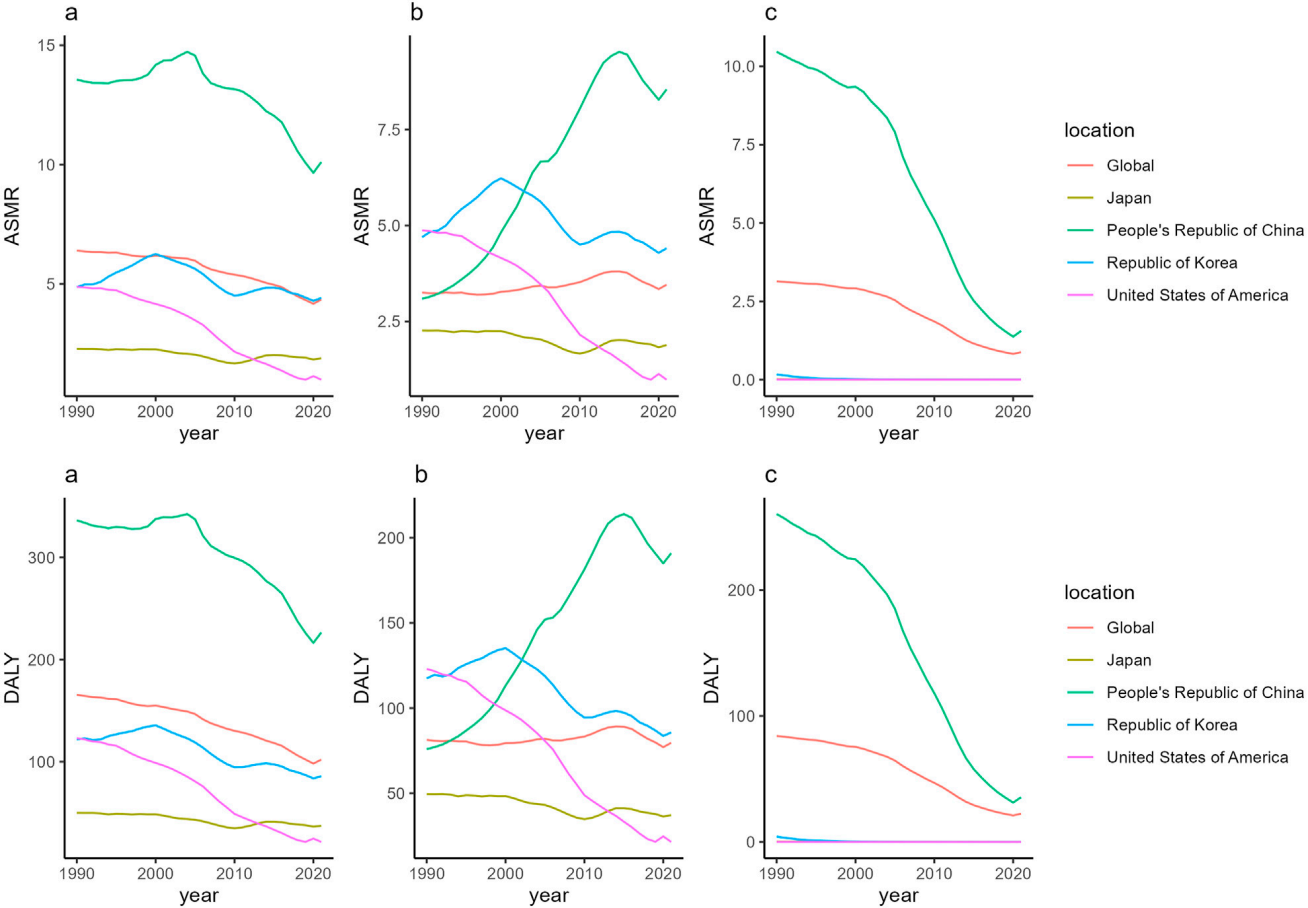
ASMR of TBL cancer attributable to PM2.5 **(a)** AP **(b)** and APM **(c)** in China, USA, Japan, Korea and global, 1990–2021; age-standardised DALY of TBL cancer attributable to PM2.5 **(a)**, HAP **(b)** and APM **(c)** in China, USA, Japan, Korea and global, 1990–2021. Study name: Study on the Burden of Tracheal, Bronchial, and Lung Cancer Related to PM2.5; Country/Region: China, the United States, Japan, South Korea and the global; Years: 1990–2021.

From 1990 to 2021, China showed a significantly higher ASMR of TBL cancer attributable to PM2.5 exposure compared to global and some countries such as Japan, South Korea, and the United States (Figure 1a). Globally, the ASMR of TBL cancer attributable to PM2.5 exposure demonstrated a downward trend with the AAPC was −1.2811 (95% CI: -1.5389 to −1.0225). Similarly, China, Japan, South Korea, and the United States also experienced decreasing trends in the ASMR ofTBL cancer attributable to PM2.5 exposure, with the AAPC were −0.9068 (95% CI: −1.5021 to −0.3078), −0.6487 (95% CI: −0.9002 to −0.3965), −0.3622 (95% CI: −0.6244 to −0.0994), and −4.9988 (95% CI: -5.5223 to −4.4724), respectively. No significant differences were observed between males and females (Table 1; Figure 1a).

**TABLE 1:** Trends in age-standardised death rate of Tracheal, bronchus, and lung cancer attributable to PM2.5, 1990–2021. Study name: Study on the Burden of Tracheal, Bronchial, and Lung Cancer Related to PM2.5; Country/Region: China, the United States, Japan, South Korea and the global; Years: 1990–2021.

Risk	Sex	China		Japan		Korea		USA		Global	
		AAPC	95% CI	AAPC	95% CI	AAPC	95% CI	AAPC	95% CI	AAPC	95% CI
Pm2.5	Both	−0.9068	(−1.5021 to −0.3078)	−0.6487	(−0.9002 to −0.3965)	−0.3622	(−0.6244 to −0.0994)	−4.9988	(−5.5223 to −4.4724)	−1.2811	(−1.5389 to −1.0225)
	Female	−0.9321	(−1.1533 to −0.7104)	−0.5206	(−0.8168 to −0.2234)	−0.2718	(−0.5683 to 0.0255)	−4.2039	(−4.7821 to −3.6223)	−0.8245	(−0.9146 to −0.7343)
	Male	−1.1255	(−1.4992 to −0.7502)	−0.8749	(−1.1037 to −0.6455)	−0.5676	(−0.8229 to −0.3117)	−5.66	(−6.1633 to −5.154)	−1.5405	(−1.8658 to −1.2141)
HAP	Both	3.3234	(2.9647–3.6833)	−0.6328	(−0.8848 to −0.3802)	−0.2804	(−0.537 to −0.023)	−4.9982	(−5.5215 to −4.472)	0.1097	(−0.1584–0.3785)
	Female	3.9766	(3.7587–4.1948)	−0.4987	(−0.7946 to −0.202)	−0.1643	(−0.4586 to 0.1309)	−4.2032	(−4.7813 to −3.6216)	1.1691	(0.9579–1.3809)
	Male	3.0238	(2.6277–3.4214)	−0.8611	(−1.0909 to −0.6307)	−0.4976	(−0.7505 to −0.244)	−5.6594	(−6.1626 to −5.1534)	−0.3264	(−0.582 to −0.0702)
APM	Both	3.3234	(2.9647–3.6833)	−0.6328	(−0.8848 to −0.3802)	−0.2804	(−0.537 to −0.023)	−4.9982	(−5.5215 to −4.472)	0.1097	(−0.1584–0.3785)
	Female	3.9766	(3.7587–4.1948)	−0.4987	(−0.7946 to −0.202)	−0.1643	(−0.4586 to 0.1309)	−4.2032	(−4.7813 to −3.6216)	1.1691	(0.9579–1.3809)
	Male	3.0238	(2.6277–3.4214)	−0.8611	(−1.0909 to −0.6307)	−0.4976	(−0.7505 to −0.244)	−5.6594	(−6.1626 to −5.1534)	−0.3264	(−0.582 to −0.0702)

^a^ Statistically significant (p < 0.05).

AAPC, average annual per cent change; APM, ambient PM; HAP, household air pollution; PM, particulate matter.

Between 1990 and 2021, the global ASMR of TBL cancer attributable to HAP exposure exhibited a stable trend (Figure 1b). However, gender-specific trends diverged significantly: the ASMR for females increased with an AAPC of 1.1691 (95% CI: 0.9579–1.3809), while that for males decreased, with an AAPC of −0.3264 (95% CI: −0.582 to −0.0702) (Table 1; Figure 1b). Notably, country-level trends in the ASMR of TBL cancer attributable to HAP exposure varied considerably. In China, the ASMR of TBL cancer attributable to HAP exposure initially increased from 1990 to 2015, then declined from 2016 to 2020, before rising again after 2020, with an overall AAPC of 3.3234 (95% CI: 2.9647–3.6833) (Table 1; Figure 1a). Conversely, Japan, South Korea, and the United States experienced consistent declines in HAP-related mortality rates, with AAPCs of −0.6328 (95% CI: −0.8848 to −0.3802), −0.2804 (95% CI: −0.537 to −0.023), and −4.9982 (95% CI: −5.5215 to −4.472), respectively (Table 1; Figure 1a).

During 1990 from 2021, the ASMR of TBL cancer attributable to APM exposure in China was significantly higher than the global average and that of countries such as Japan, South Korea, and the United States (Figure 1c). Globally, the ASMR of TBL cancer attributable to APM exposure exhibited a declining trend, with an AAPC of 0.1097 (95% CI −0.1584–0.3785) (Table 1; Figure 1c). In contrast, the ASMR of TBL cancer attributable to APM exposure in China also decreased, with an AAPC of 3.3234 (95% CI 2.9647–3.6833) (Table 1; Figure 1c). However, the ASMR of TBL cancer attributable to APM exposure in Japan, South Korea, and the United States remained relatively stable during this period (Table 1; Figure 1c).

**FIGURE 2:**
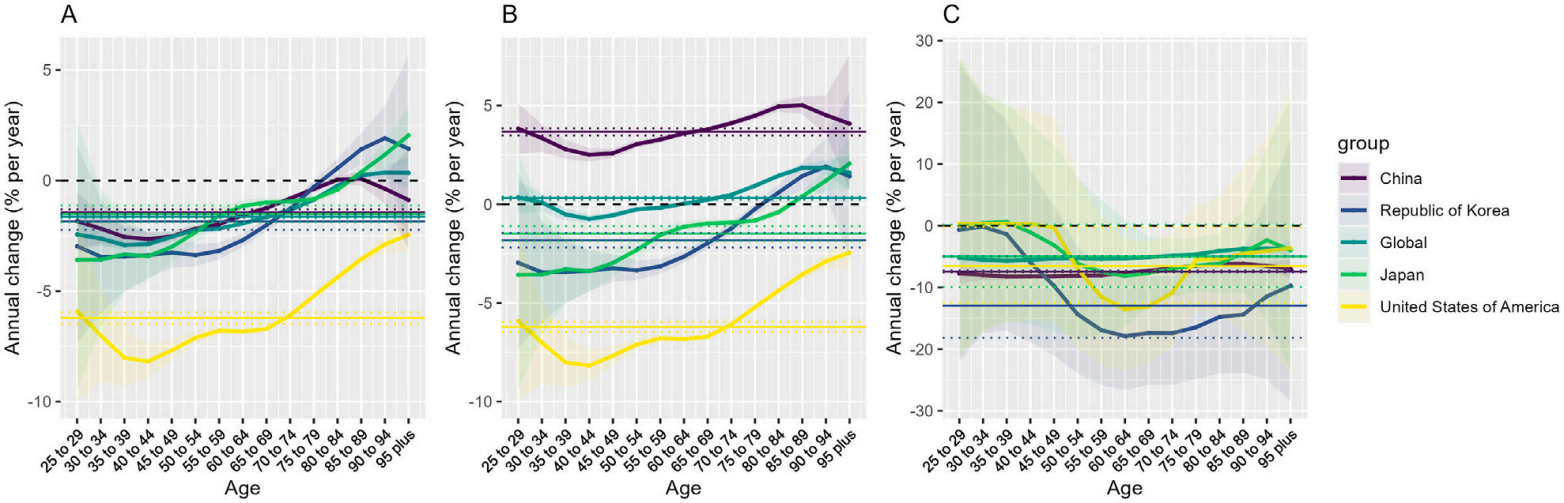
Net and local drift values of TBL cancer mortality attributable to particulate matter2.5 **(A)** household air pollution **(B)** and ambient particulate matter **(C)** in China, USA, Japan, Korea and global, 1990–2021. Study name: Study on the Burden of Tracheal, Bronchial, and Lung Cancer Related to PM2.5; Country/Region: China, the United States, Japan, South Korea and the global; Years: 1990–2021.

Between 1990 and 2021, the ASMR of TBL cancer attributable to PM2.5 exposure in China was significantly higher than the global average and that of countries such as Japan, South Korea, and the United States (Figure 2A). Globally, the ASMR of TBL cancer attributable to PM2.5 exposure exhibited a declining trend, with an AAPC of −1.5967 (95% CI: −1.8294 to −1.3635). Similarly, the ASMR of TBL cancer attributable to PM2.5 exposure in China, Japan, South Korea, and the United States also showed downward trends, with AAPC of −1.2483 (95% CI: -1.7432 to −0.751), −1.0024 (95% CI: −1.2659 to −0.7383), −5.44 (95% CI: −5.9458 to −4.9314), and −5.44 (95% CI: −5.9458 to −4.9314), respectively. Notably, there was no significant difference in these trends between males and females (Table 2; Figure 2A).

**TABLE 2:** Trends in age-standardised disability-adjusted life year of Tracheal, bronchus, and lung cancer attributable to PM2.5,1990–2021. Study name: Study on the Burden of Tracheal, Bronchial, and Lung Cancer Related to PM2.5; Country/Region: China, the United States, Japan, South Korea and the global; Years: 1990–2021.

Risk	Sex	China		Japan		Korea		USA		Global	
		AAPC	95% CI	AAPC	95% CI	AAPC	95% CI	AAPC	95% CI	AAPC	95% CI
Pm2.5	Both	−1.2483	(−1.7432 to −0.751)	−1.0024	(−1.2659 to −0.7383)	−1.1483	(−1.2456 to −1.0526)	−5.44	(−5.9458 to −4.9314)	−1.5967	(−1.8294 to −1.3635)
	Female	−1.2721	(−1.4751 to −1.0687)	−0.9203	(−1.23 to −0.6097)	−0.9710	(−1.0833 to −0.8672)	−4.6958	(−5.2491 to −4.1393)	−1.1303	(−1.2197 to −1.0409)
	Male	−1.4238	(−1.77 to −1.0764)	−1.2178	(−1.4707 to −0.9643)	−1.3553	(−1.4585 to −1.2570)	−6.0165	(−6.5064 to −5.524)	−1.8319	(−2.1219 to −1.5411)
HAP	Both	3.0044	(2.6674–3.3425)	−0.9867	(−1.2506 to −0.7222)	−1.0683	(−1.3214 to −0.8144)	−5.4394	(−5.9451 to −4.931)	−0.1675	(−0.4255 to 0.0912)
	Female	3.687	(3.4826–3.8918)	−0.899	(−1.2094 to −0.5875)	−0.8645	(−1.1459 to −0.5823)	−4.6953	(−5.2483 to −4.1391)	0.9509	(0.6613–1.2413)
	Male	2.7462	(2.3729–3.1209)	−1.2046	(−1.4594 to −0.949)	−1.2856	(−1.5401 to −1.0305)	−6.0159	(−6.5057 to −5.5235)	−0.5765	(−0.8177 to −0.3346)
APM	Both	3.0044	(2.6674–3.3425)	−0.9867	(−1.2506 to −0.7222)	−1.0683	(−1.3214 to −0.8144)	−5.4394	(−5.9451 to −4.931)	−0.1675	(−0.4255 to 0.0912)
	Female	3.687	(3.4826–3.8918)	−0.899	(−1.2094 to −0.5875)	−0.8645	(−1.1459 to −0.5823)	−4.6953	(−5.2483 to −4.1391)	0.9509	(0.6613–1.2413)
	Male	2.7462	(2.3729–3.1209)	−1.2046	(−1.4594 to −0.949)	−1.2856	(−1.5401 to −1.0305)	−6.0159	(−6.5057 to −5.5235)	−0.5765	(−0.8177 to −0.3346)

^a^ Statistically significant (p < 0.05).

AAPC, average annual per cent change; APM, ambient PM; HAP, household air pollution; PM, particulate matter.

From 1990 to 2021, the global ASMR of TBL cancer attributable to HAP exposure exhibited a stable trend (Figure 2B). However, the ASMR of TBL cancer attributable to HAP for females showed an upward trend, with an AAPC of 0.9509 (95% CI: 0.6613–1.2413), while that for males showed a downward trend, with an AAPC of −0.5765 (95% CI: -0.8177 to −0.3346) (Table 2; Figure 2B). Notably, the trends in the ASMR of TBL cancer attributable to HAP exposure varied significantly among different countries. In China, the ASMR of TBL cancer attributable to HAP exposure increased from 1990 to 2015, decreased from 2016 to 2020, and then rose again after 2020, with an overall AAPC of 3.0044 (95% CI: 2.6674–3.3425) (Table 2; Figure 2B). In contrast, Japan, South Korea, and the United States experienced consistent declines in ASMR of TBL cancer attributable to HAP exposure, with AAPC of −0.9867 (95% CI -1.2506 to −0.7222), −1.0683 (95% CI -1.3214 to −0.8144), and −5.4394 (95% CI -5.9451 to −4.931), respectively (Table 2; Figure 2B).

Between 1990 and 2021, the ASMR of TBL cancer attributable to APM exposure in China was significantly higher than the global average and that of countries such as Japan, South Korea, and the United States (Figure 2C). Globally, the ASMR of TBL cancer attributable to APM exposure exhibited a downward trend, with an AAPC of −0.1675 (95% CI: -0.4255 to 0.0912) (Table 2; Figure 2C). Similarly, the ASMR of TBL cancer attributable to APM exposure also showed a downward trend, with an AAPC of 3.0044 (95% CI 2.6674–3.3425) (Table 2; Figure 2C). However, the ASMR of TBL cancer attributable to APM exposure in Japan, South Korea, and the United States remained stable during this period (Table 2; Figure 2C).

### Ambient PM-Attributable TBL Cancer APC Mortality Trends (1990–2021)

**FIGURE 3:**
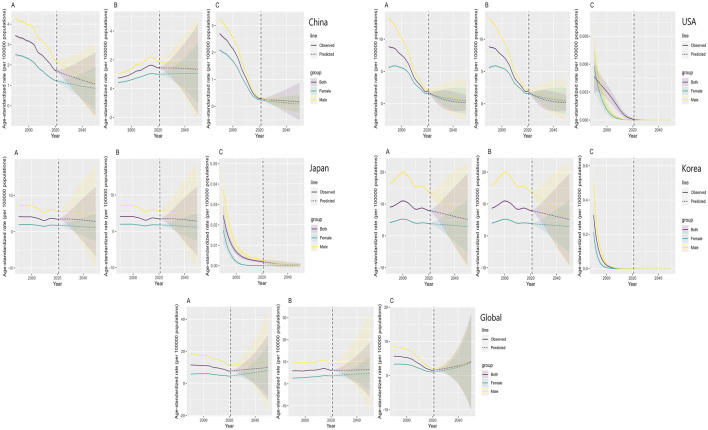
BAPC Analysis of TBL cancer mortality attributable to particulate matter2.5 **(A)** household air pollution **(B)** and ambient particulate matter **(C)** in China, USA, Japan, Korea and global, 1990–2050.

From 1990 to 2021, the mortality burden of TBL cancer attributable to PM2.5 has shown a decreasing trend across all age groups in China and the United States. Globally and in Japan, this trend is observed for individuals aged 25–89, while there has been an increase in the mortality burden for those aged 89 and older. In South Korea, the mortality burden TBL cancer attributable to PM2.5 has decreased among individuals aged 25–79 but has increased for those aged 79 and older (Table 3; Figure 3A). In addition, the mortality burden of TBL cancer attributable to HAP decreased across all age groups in the United States, whereas it increased across all age groups in China. On a global scale, in Japan and South Korea, the mortality burden of TBL cancer attributable to HAP exposure decreased among younger age groups but increased among older age groups (Table 3; Figure 3B). However, the global mortality burden of tracheal, bronchus, and lung cancer attributable to APM has shown a decreasing trend across all age groups, including in major countries such as China, the United States, Japan, and South Korea (Table 3; Figure 3C).

**TABLE 3:** Age-period-cohort analysis of Tracheal, bronchus, and lung cancer mortality attributable to PM from 1990 to 2019. Study on the Burden of Tracheal, Bronchial, and Lung Cancer Related to PM2.5, China, the United States, Japan, South Korea and the global, 1990 -2021.

Item	China			Japan			Korea			USA			Global		
	PM2.5	APM	HAP	PM2.5	APM	HAP	PM2.5	APM	HAP	PM2.5	APM	HAP	PM2.5	APM	HAP
Net drift = 0	412.83	1,642.51	11,724.06	60.36	58.86	3.58	95.03	92.34	19.44	2,104.47	2,103.78	4.11	2,967.64	56.80	11,299.59
All local drifts = net drift	407.15	215.38	379.59	184.06	182.97	1.69	246.34	244.83	4.619	857.62	857.59	3.73	1720.73	662.08	304.86
All period RR = 1	670.49	2,190.96	13,829.79	234.90	231.89	6.24	136.98	135.84	13,829.79	2,602.71	2,602.62	4.19	3,184.44	224.56	12,740.08
All cohort RR = 1	1,273.05	5,762.17	31,211.34	304.12	298.93	16.55	468.62	455.15	25.36	15,360.50	15,351.87	8.53	7,990.78	706.25	27,003.08
All age deviations = 0	2,781.49	2,624.94	3,243.12	1,119.26	1,118.54	2.39	479.43	475.96	2.32	4,291.22	4,286.64	2.17	22,346.13	12,289.28	11,540.96
All period deviations = 0	201.02	896.49	3,523.37	186.44	184.75	2.12	44.52	45.41	1.36	570.28	570.82	0.44	113.86	224.56	2,106.25
All cohort deviations = 0	415.29	229.86	381.18	186.83	185.89	2.27	257.17	255.55	5.00	859.82	859.82	4.04	1774.77	706.25	315.15

^a^ Statistically significant (p < 0.05).

APM, ambient PM; HAP, household air pollution; PM, particulate matter; RR, relative risk.

### Projected Global Burden of TBL Cancer From Ambient PM Pollution (2022–2050)

Based on the BAPC model predictions, we found that the global ASMR of TBL cancer attributable to PM2.5 exposure is projected to increase over the next 29 years. The core assumptions of our prediction model are: (a) Pollution trend continuity: Extrapolated from 1990 to 2021 historical concentration change rates of PM2.5, HAP, and APM across countries (China’s 0.9% annual PM2.5 reduction, U.S. 4.9%; GBD 2021 data), assuming current air pollution control policies persist [11]. (b) Demographic parameters: Incorporating UN 2022 population projections (age structure, gender ratio), with stable cohort effect impacts on cancer risk [8]. (c) Model priors: Weakly informative priors (normal for period/cohort effects, mean = 0, SD = 1); posterior distributions estimated via INLA, converging at PSRF <1.01. 95% confidence intervals (CI) from posterior sampling reflect parameter uncertainty [14].

However, the ASMR in China, South Korea, and the United States is expected to show a significant decrease, while Japan’s trend is anticipated to remain stable. Similarly, the global ASMR of TBL cancer attributable to HAP exposure is also projected to increase over the next 29 years. In China, the ASMR due to HAP exposure is expected to rise, whereas in South Korea, Japan, and the United States, it is forecasted to significantly decrease. Furthermore, the global ASMR of TBL cancer attributable to APM exposure is projected to increase over the next 29 years. Conversely, the ASMR in China, South Korea, Japan, and the United States is expected to show a significant decrease (Figure 3).

## Discussion

### Main Findings and Significance

This study utilized the 2021 Global Burden of Disease (GBD) data to examine global and regional trends in the burden of TBL cancers attributable to PM2.5 exposure across China, the United States, Japan, and South Korea from 1990 to 2021. The findings revealed significant disparities, with China consistently exhibiting higher age-standardized mortality rates (ASMR) and age-standardized disability-adjusted life years (ASDR), reflecting the compounded health burden associated with industrialization and urbanization [15]. Notably, the burden of disease linked to household air pollution (HAP) in China displayed fluctuating trends, contrasting with the more consistent declines observed in other nations. This variation can likely be attributed to the ongoing shifts in energy structures between urban and rural regions in China, as well as differences in policy enforcement [16, 17].

In comparison, the United States showed the most pronounced reduction in ASMR and ASDR, which can be attributed to effective regulatory frameworks, such as stringent air quality standards and advances in clean energy technologies. However, projections from the Bayesian Age-Period-Cohort (BAPC) model suggest an alarming global trend: the ASDR attributable to PM2.5 exposure is expected to rise over the next 29 years. This increase may be driven by regional disparities in pollution control, the exacerbation of aging populations, and gaps in policy implementation and economic development in certain regions [18, 19]. Specifically, based on the BAPC model’s assumption of continuous pollution trends (extrapolated from 1990 to 2021 historical concentration change rates), regional differences in pollution control efficacy play a dominant role. For instance, if PM2.5 concentrations in low- and middle-income regions continue to increase at the current annual rate of 1.2% (consistent with GBD 2021 data), their contribution to the global ASDR rise could reach 60%–70%. In contrast, high-income countries, with a 4.9% annual reduction in PM2.5, may offset approximately 30% of the global increase. This aligns with our observation that China’s PM2.5-related ASMR remains significantly higher than that of the United States, underscoring how delayed pollution control measures act as a core driver of rising ASDR. These findings underscore the urgent need for enhanced global cooperation and tailored, region-specific interventions [3].

### Gender and Age-Specific Patterns

While the study revealed minimal overall gender differences in the PM2.5-related disease burden, a rising burden was noted among women, particularly related to HAP. This trend is likely linked to the higher exposure of women to traditional biomass fuels during domestic activities, especially in rural areas [20]. Physiological and behavioral factors, such as smoking rates and physical activity levels, may further contribute to these gender disparities [21]. There is a significant synergistic effect between PM2.5 exposure and smoking: PM2.5 can enhance the bioavailability of carcinogens in tobacco smoke, while smoking-induced damage to the respiratory mucosa increases the body’s susceptibility to harmful substances in PM2.5. The risk of combined exposure to both factors is much higher than that of exposure to either factor alone [22]. This interaction may lead to either an underestimation or overestimation of the PM2.5-attributable burden of tracheal, bronchial, and lung (TBL) cancers in this study, especially in countries with high smoking rates (the smoking rate is approximately 50.5% among men and 2.1% among women in China, and about 12% among men and 9.1% among women in the United States) [23, 24].

Age-stratified analysis indicates that the disease burden in the elderly population is significantly increased, which is closely associated with long-term cumulative exposure to PM2.5. [25, 26]. In terms of disease pathogenesis, PM2.5-related diseases (such as TBL cancers) typically have a long latency period (the latency period of TBL cancers is mentioned in the text to be generally 20–30 years). Therefore, the manifestation of diseases in the elderly is largely a result of long-term exposure to PM2.5 in the early stages of life, a process analogous to the delayed onset of smoking-related lung cancer — risk exposure occurs at an earlier stage, while disease manifestations are more common in later life [27, 28].

This trend was particularly evident in China and South Korea, where rapid population aging amplifies the health impacts of air pollution [29, 30]. Specifically, according to UN 2022 population projections, the global proportion of people aged 65 and above is projected to rise from 10% in 2021 to 22% by 2050. This demographic shift, by amplifying the cumulative exposure effects of PM2.5, may contribute 20%–25% of the projected increase in ASDR. For instance, in Japan, each 1% increase in the aging rate has been associated with a 0.8% rise in PM2.5-related lung cancer mortality (consistent with the age-period-cohort analysis results in Figure 3A) [31, 32]. These findings emphasize the need for age-specific public health interventions, such as improving access to healthcare for older populations, promoting clean energy alternatives, and launching educational campaigns aimed at vulnerable groups.

### Inter-Country Differences and Policy Implications

The significant inter-country differences observed in this study underscore the varying effectiveness of air pollution control strategies. The United States’ notable progress in reducing the disease burden attributable to PM2.5 is largely attributed to robust policy measures, including the Clean Air Act, extensive health monitoring, and the promotion of clean energy solutions [33]. In contrast, Japan and South Korea, given their lower baseline pollution levels, have exhibited slower declines in PM_2_._5_-related TBL cancer burden. This trend likely reflects the “diminishing returns” in pollution control: when ambient PM_2_._5_ concentrations are already close to or below stringent environmental standards, the absolute reduction potential becomes limited, and achieving further declines requires disproportionately higher technological and economic investments. Notably, the slower trends here refer to percentage declines in age-standardized mortality rates (ASMR), as their absolute PM_2_._5_ concentration reductions (in μg/m^3^) have remained modest due to the low baseline—consistent with global observations that pollution control efficacy weakens as concentrations approach background levels. [8, 32].

China’s high disease burden highlights the compounded effects of rapid industrialization, urbanization, and imbalanced energy structures, particularly in rural areas. Although recent policies such as the “Blue Sky Protection Campaign” have yielded some improvements, the Beijing-Tianjin-Hebei urban agglomeration has achieved pollution mitigation through targeted control models [34], while changes in air pollution across multiple countries during the COVID-19 pandemic have also provided a reference for evaluating policy effectiveness [35]. Inconsistent enforcement and the urban-rural divide in the adoption of clean energy continue to pose significant challenges [33, 36]. The fluctuating trends in HAP-related disease burdens further underscore the critical need for sustained and comprehensive policy implementation, particularly in rural regions.

Beyond policy frameworks, other factors contribute disproportionately to cross-national disparities. With respect to energy structures, household air pollution (HAP) remains a hidden driver: in developing countries where clean energy transitions stall, HAP could account for an additional 15%–20% increase in female ASDR (consistent with the rising trend of female HAP-related ASDR in Figure 2B). In contrast, HAP contributes less than 5% to overall disease burden in high-income countries, rendering its impact negligible. Meanwhile, healthcare capacity plays a buffering role: high-income nations, such as the United States, have reduced ASDR by 5%–8% through improvements in 5-year survival rates for PM2.5-related cancers (now exceeding 60%) [37]. Low-income regions, however, lack such healthcare resources, eliminating this protective effect and exacerbating ASDR increases. These nuances highlight that addressing air pollution-related disease burdens requires not only pollution control policies but also targeted interventions in energy transition and healthcare equity [38].

### Integration of Multifactorial Contributions and Links to Previous Research

A comprehensive analysis reveals hierarchical drivers behind the projected global rise in PM2.5-related age-standardized death rate (ASDR): (a) Primary drivers (accounting for over 80% of the increase) [1]: Lagging pollution control, contributing 60%–70%, with the most severe impacts in low- and middle-income regions due to weak regulation and industrial expansion [2]; Population aging, contributing 20%–25%, particularly acute in countries like China and South Korea, where rapid demographic shifts amplify risks from long-term PM2.5 exposure. (b) Secondary factors (exacerbating regional disparities): Delayed energy structure transitions (10%–15% contribution), especially persistent reliance on solid fuels in rural developing areas, which exacerbate burdens on vulnerable groups like women; conversely, healthcare capacity in high-income countries mitigates ASDR (−5% to −8% contribution) through advanced screening and treatment. (c) This framework highlights the need to prioritize cross-border pollution governance (especially in low- and middle-income regions), alongside targeted health protections for the elderly and accelerated clean energy transitions, to counteract overlapping risks.

### Integration of Multifactorial Contributions and Links to Previous Research

This study, as at comprehensive burden of disease assessment, quantifies and projects the long-term trends in PM2.5-attributable TBL cancer mortality and DALYs across four major economies from 1990 to 2050. Our findings of declining age-standardized rates (ASMR/ASDR) in most study countries over the past three decades align with and extend the evidence base established by recent large-scale burden assessments [8], such as analyses highlighting global reductions in PM2.5-attributable mortality. For example, those analyses that highlight the global decline in PM2.5-related mortality, as well as other literatures focusing on the analysis of cancer risk factors [8, 9].

However, our analysis provides distinct contributions by:

a. Directly comparing trends across four key industrialized nations (China, US, Japan, South Korea) with divergent socioeconomic development and pollution control trajectories, revealing significant disparities in burden levels and reduction rates.
b. Decomposing the contributions of household (HAP) and ambient (APM) particulate matter pollution sources to the overall PM2.5-attributable burden, uncovering divergent trends (e.g., rising female HAP burden globally vs. declining APM burden).
c. Employing age-period-cohort (APC) analysis to disentangle the effects of aging, temporal changes, and birth cohort influences on mortality trends, providing deeper insights into the drivers behind observed patterns (e.g., the amplified burden in aging populations).
d. Projecting future burdens to 2050 using Bayesian APC modeling, highlighting an anticipated concerning *global increase* in ASDR despite projected declines in most study countries, underscoring the need for intensified global action.

Furthermore, the stark inter-country differences observed, particularly China’s persistently higher burden compared to the US’s significant reductions, offer a valuable comparative lens for evaluating the real-world effectiveness of different air pollution governance strategies and healthcare system capacities over an extended period.

Despite declining ASDR in some nations, projected global increases highlight the need for continuous monitoring and adaptive policymaking. Challenges like population aging, growing energy demands, and urbanization in low- and middle-income countries require robust, flexible policy responses.

### Limitations

This study has several limitations. First, the Global Burden of Disease model’s estimates are fundamentally reliant on the availability and quality of underlying data sources considering across vital registration systems, cancer registries, household surveys, pollution monitoring While the US, Japan, and South Korea typically have mature national monitoring systems, data coverage and quality in certain regions of China—particularly historically and in rural areas—may pose challenges, potentially compromising the precision and comparability of estimates, for instance household air pollution (HAP)-related burden, across periods, such inherent data heterogeneity could introduce bias or uncertainty in cross-country comparisons and trend assessments [39, 40]. Second, the analysis focuses solely on the disease burden attributable to PM2.5 exposure, without accounting for potential synergistic or antagonistic effects with other prevalent air pollutants—including ozone, nitrogen dioxide, sulfur dioxide, and polycyclic aromatic hydrocarbons [41], Finally model Assumptions and Scope: Like all models, the GBD framework and APC/BAPC analyses employed herein rely on specific assumptions and methodologies. While these assumptions and methodologies are fairly rigorous, they still include suppositions regarding exposure-response relationships, counterfactual exposure levels, and risk factor interactions. This premise may be invalidated in the event of significant and unforeseen policy shifts, technological breakthroughs, or socioeconomic disruptions in any country [42].

### Future Research Directions

To enhance the understanding of PM2.5-related health impacts and inform more effective policy interventions, future research should prioritize the following areas a.

a. Detailed Source Attribution and Sectoral Contributions

It is crucial to conduct high-resolution assessments of pollution sources to quantify the relative contributions of various sectors, such as industrial emissions, transportation, and residential energy consumption, to the overall health burden. Such studies should aim to identify the most significant sources of PM2.5 exposure within different contexts and regions, enabling targeted interventions that address sector-specific contributions to air quality deterioration.

b. Comprehensive Long-Term Economic and Health Impact Analysis

Future investigations should explore the long-term cost-benefit dynamics of different air pollution governance strategies. By examining the economic implications alongside health outcomes, researchers can develop frameworks that optimize policy effectiveness while considering the trade-offs between environmental regulation, public health investments, and economic growth. This type of analysis would be instrumental in guiding decision-making, particularly in countries with limited resources and competing development priorities.

c. Tailored Health Interventions for Vulnerable Populations

Given the disproportionate burden of PM2.5-related health risks on vulnerable groups, such as rural women and the elderly, future research should focus on the development and evaluation of targeted health interventions. These interventions should be designed with a deep understanding of the socio-economic, behavioral, and environmental factors that contribute to heightened exposure and susceptibility in these populations. Specific strategies may include improving access to clean cooking technologies, enhancing public health education, and fostering community-based interventions that address local needs.

### Conclusion and Policy Recommendations

This study highlights the critical role of air pollution control in reducing the disease burden of TBL cancers attributable to PM2.5 exposure. While some countries have made significant progress, the projected global increase in disease burden underscores the need for stronger international collaboration, enhanced governance strategies, and targeted interventions. Policymakers should prioritize comprehensive and equitable energy transitions, strengthen health systems to protect vulnerable populations, and continuously adapt policies to emerging challenges such as aging populations and economic disparities. Persistent monitoring and scientific research will be vital in ensuring the effectiveness of these measures and advancing global health outcomes.
